# The current status of uptake of European Basic Safety Standard (2013/59/Euratom) requirements: results of a follow-up survey in European radiology departments

**DOI:** 10.1186/s13244-021-01078-3

**Published:** 2021-10-07

**Authors:** European Society of Radiology (ESR), David C. Howlett, Adrian P. Brady, Nuria Bargalló, Guy Frija, Steve Ebdon-Jackson, Alexandra Karoussou-Schreiner

**Affiliations:** Am Gestade 1, Vienna, Austria

**Keywords:** Clinical audit, European Commission, QuADRANT, SAMIRA, Radiation protection

## Abstract

The recently implemented European Council Basic Safety Standards Directive (BSSD), 2013/59/Euratom lays down core radiation protection standards for European radiology departments, including a mandatory requirement for supporting processes of clinical audit. A repeat survey on behalf of the European Society of Radiology (ESR) was undertaken in February 2021, involving the ESR EuroSafe Imaging Star department network, to re-assess compliance with selected key BSSD requirements following an initial survey in 2018 where variable compliance was demonstrated. 61% (78/128) of eligible departments participated and overall the survey results revealed a mixed picture in terms of implementation of BSSD requirements when compared to the 2018 survey with both improvement and deterioration observed. This pattern was seen also in relation to supporting processes of regulatory audit and re-audit. Higher levels of “skipping” of responses were also observed in 2021. These findings were unexpected in light of the interventions in relation to audit (clinical and regulatory) and radiation protection undertaken by the ESR and other organisations in recent years, but can reasonably be explained by the onset of the COVID-19 pandemic, with consequent significant disruption of radiology services. The 2021 survey results do serve to highlight again the need for co-ordinated intervention involving relevant European bodies, organisations and governmental agencies to address the important issues raised by this survey. The European Commission clinical audit and radiation protection initiatives, QuADRANT, led by the ESR, and SAMIRA will act as important drivers for improvement in patient safety, experience and outcomes across Europe.

**Supplementary Information:**

The online version contains supplementary material available at 10.1186/s13244-021-01078-3.

## Key points

- The European Council Basic Safety Standards Directive (BSSD), 2013/59/Euratom lays down legally required standards for radiation protection.
- Clinical audit is a mandatory component of the BSSD, radiology departments will be required to show evidence of compliance with the BSSD and supporting processes of clinical audit.
- A repeat survey on behalf of the ESR was undertaken in 2021 amongst the ESR EuroSafe Imaging Star network departments, re-assessing BSSD compliance following a 2018 survey.
- 61% of departments participated. Survey results demonstrated a mixed picture of compliance with BSSD radiation protection and clinical audit requirements when compared to 2018, the COVID-19 pandemic is likely to have been influential.
- The survey results indicate the need for co-ordinated pan-European action. The European Commission initiatives, QuADRANT, led by the ESR, and SAMIRA will help facilitate necessary improvements in implementation of radiation protection and clinical audit requirements.

## Patient Summary

The European Council Basic Safety Standards Directive (BSSD), 2013/59/Euratom is an important piece of legislation, laying down standards for radiation protection and for transposition into the legislation of European Union Member States by February 2018. The BSSD specifically mandates the need for supporting processes of clinical audit “according to national procedures”. Implementation of BSSD requirements therefore is an area of priority for European Radiology departments.

Clinical audit is a process involving systematic review of practices, procedures and results against agreed standards, with modification of practices as needed and the application of new standards where necessary. Clinical audit is recognised as an important component of clinical governance and has a key role in improving patient care, experience and outcomes.

This paper describes the results of a follow-up survey amongst European radiology departments, following an original survey in 2018, which has demonstrated variable compliance with BSSD implementation and supporting audit processes. Despite a variety of radiation protection and audit-related initiatives, introduced via the ESR and other organisations, the repeat survey demonstrated a mixed picture of improvement and deterioration in compliance when compared to the 2018 survey.

The results were unexpected but can be explained by the devastating COVID-19 pandemic that has had a significant impact and many radiology departments have struggled to maintain staffing and service levels. The current survey does highlight the ongoing need for the ESR, working in collaboration with other national and professional societies and governmental bodies, to continue to promote clinical audit and to support departments in implementation of BSSD requirements. Important European Commission initiatives, QuADRANT (led by the ESR) and SAMIRA will help drive improvements in patient care across Europe.

## Introduction

The European Council Basic Safety Standards Directive (BSSD), 2013/59/Euratom [1], laying down the requirements for protection from the dangers associated with medical ionising radiation exposure, was adopted by the Council of the European Union (EU) in 2013, for transposition into the national legislation of EU Member States by February 2018. The BSSD, through national implementing legislation, directly impacts on all radiology departments and contains several key updated aspects in light of new scientific evidence and guidelines - these are summarised in a recent ESR publication [2]:

- Changes in justification processes.
- Consistent use and monitoring of DRLs (diagnostic reference levels).
- More stringent requirements around recording and reporting doses arising from radiological procedures.
- Changes in patient information requirements.
- New dose limits for the eye and for occupational and student exposure.
- Clarification of the role of the medical physics expert.

The BSSD also specifically highlights the need for radiology departments to carry out clinical audit “in accordance with national procedures”, thereby allowing a degree of flexibility according to national resources and audit infrastructures, but making clear that supporting clinical audit activity is mandatory and a legal requirement.

Regulatory audit, whilst not clinical audit as envisioned within the BSSD, is a type of audit that verifies compliance with regulation and standards [3]. The ESR considers it important for radiology departments to develop effective regulatory audit processes – to ensure and improve patient safety, to confirm that departments are complying with BSSD requirements and also to give confidence that departments can satisfy the relevant competent authority during inspections.

In order to ascertain European radiology departmental compliance with BSSD requirements the ESR undertook a survey, distributed to all departments within the EuroSafe Imaging Star Network, in late 2018 [4]. The survey examined departmental implementation of a range of key BSSD radiation protection requirements [2], also evaluating supporting processes of departmental regulatory audit and re-audit for each requirement. The emphasis on implementation of specific BSSD requirements and supporting departmental regulatory audit to ensure continuing compliance was deliberate and felt to be the most immediate priority at the time for European radiology departments. Only a brief and general question on supporting clinical audit infrastructure was included.

The survey response rate was good (64%) and showed variable levels of compliance. Responses confirmed 100% compliance with only one parameter (the ability of CT equipment to record patient dose), 100% compliance was not recorded for supporting regulatory audit/re-audit processes. Questions around justification, dose limits for workers and patient information revealed relatively low levels of compliance.

The survey results were felt likely to be representative of wider European radiological practice and would require targeted and co-ordinated multi-agency co-operation and intervention to facilitate necessary improvements. The ESR has introduced a number of radiation protection and clinical and regulatory audit-related initiatives before and since the 2018 survey (these are covered in detail in the discussion). A re-survey was proposed to allow an assessment to be made of any positive effects arising from ESR (and other agency) initiatives and whether improvements in BSSD compliance could be observed. This paper describes the results of the 2021 re-survey of European radiology departments and discusses their implications.

## Materials and methods

The survey was prepared by members of the ESR Audit and Standards sub-committee, the ESR EuroSafe Imaging Steering Committee and the ESR Office. The questionnaire was created using Survey Monkey, with similar format to the 2018 survey, allowing ease of completion, return and analysis of results.

The questionnaire was an abbreviated version of the original survey, containing a total of 14 questions (the original 2018 survey comprised 22 questions). Questions were included covering topics where compliance was noted to be low in the 2018 survey (the questions are included in Table 2 and Additional file 1), the emphasis again being on BSSD requirement implementation and supporting regulatory audit. The majority of questions were structured in the same way as utilised in 2018, with 3 components: -

1. Has the requirement in question been implemented in your department?
2. Does the department have a programme in place to audit the requirement?
3. Is regular re-audit carried out or planned?

As previously it was proposed to distribute the survey to the ESR EuroSafe Imaging Star network. These departments will have met the existing requirements to join the network, including those relating to radiation protection (based upon the EuroSafe Imaging 2018 Call for Action [5]). A proportion of departments are likely to have acquired EuroSafe Imaging Star status prior to the BSSD and its transposition and there have also been changes in Imaging Star status requirements since 2018, so potentially there may be a degree of variation in actual departmental BSSD implementation. It is also important to note that a proportion of EuroSafe Imaging Star departments are outside of the EU, and are therefore not legally bound by the BSSD, their results are however included.

The survey was launched to all EuroSafe Imaging Star radiology departments on the ESR database at the beginning of February 2021. An initial closing date for data submission was proposed after 4 weeks, a 2-week extension was then provided, with the survey completing on 17^th^ March 2021. As part of the process the anonymity of respondents was guaranteed.

## Results

At the time of survey closure responses to the questionnaire had been received from 78 out of a potential 128 departments (61%).

Table 1 includes all those countries who have ≥ 1 EuroSafe Imaging Star department and also the number of departments in that country who responded, 2018 response rates by country are also included. Note that two countries (Switzerland and Turkey) are non-EU members but they have EuroSafe Imaging Star departments and they are included in the survey.

The results of the 2021 survey (alongside the questions) are shown in Table 2 and Additional file 1. Table 2 contains a question covering the availability of written processes informing key persons/agencies of significant accidental radiation exposure, the answers are separated for ease of review.

**Table 1:**
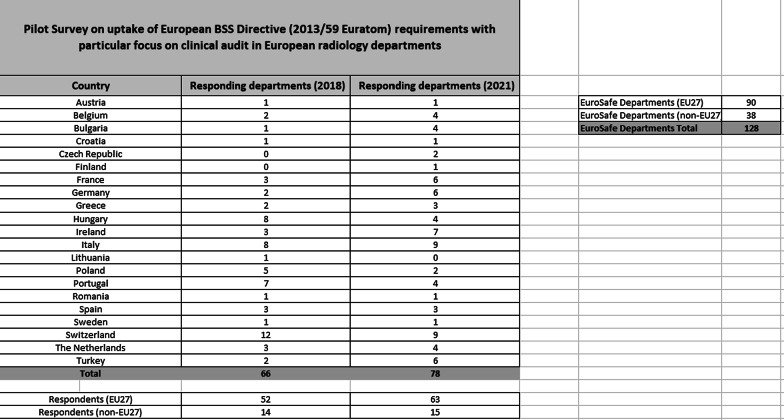
Pilot Survey on uptake of European BSS Directive (2013/59 Euratom) requirements with particular focus on clinical audit in European radiology departments

**Table 2:**
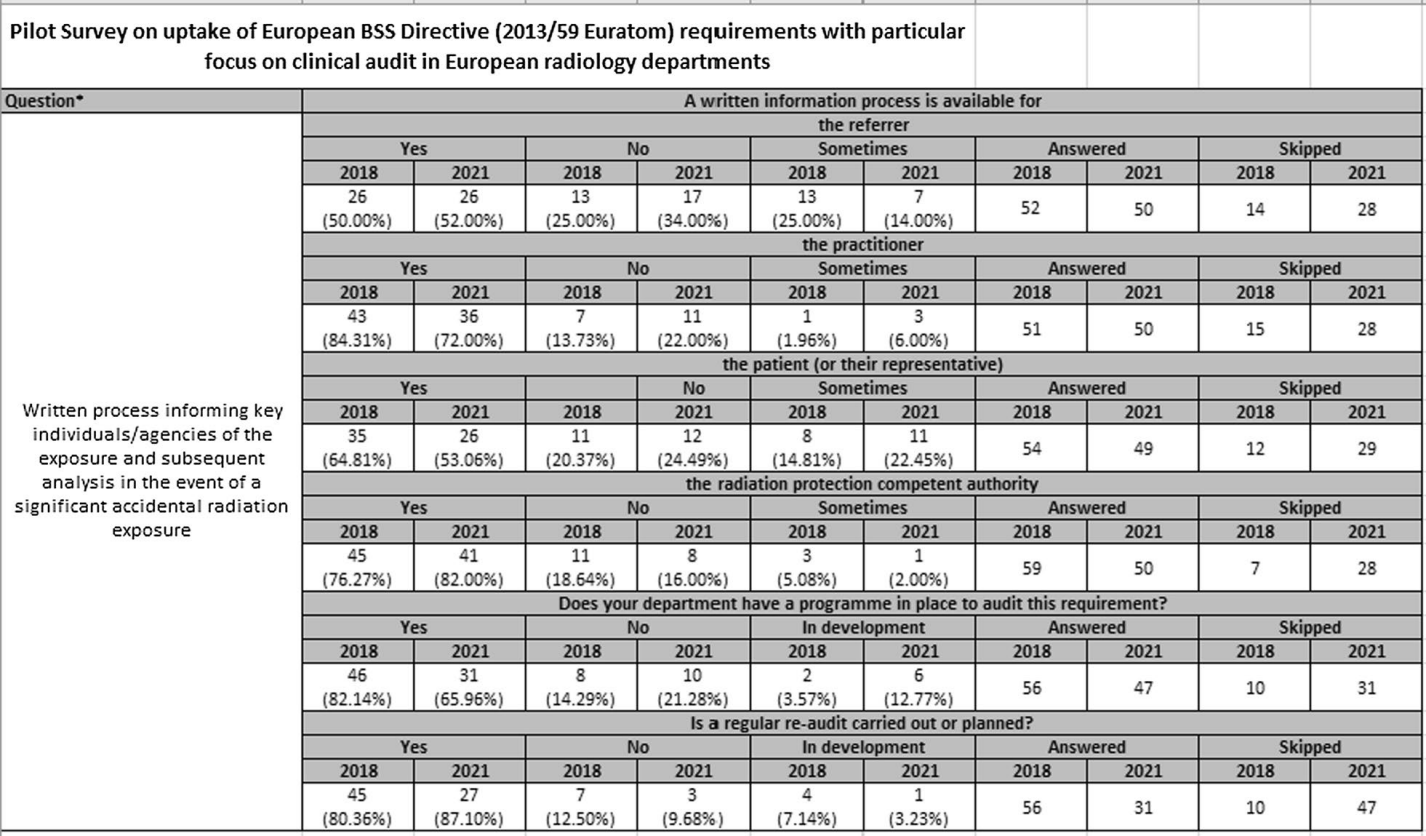
Pilot Survey on uptake of European BSS Directive (2013/59 Euratom) requirements with particular focus on clinical audit in European radiology departments

Comparison results from the 2018 survey are also included - “Yes” and “No” responses for both the 2021 and 2018 surveys have been worked out using the total number of “Yes” and “No” responses divided by the total “Yes/No” number of respondents to that question giving a % response rate; it is this figure that is used to evaluate relative differences in compliance between the surveys. “Skipped” responses are also recorded.

The results themselves are discussed in more detail in the discussion section.

## Discussion

The European Council Basic Safety Standards Directive (BSSD), 2013/59/Euratom is an extremely important piece of legislation and became effective in EU Member States as of February 2018. European Member States are required to transpose its requirements into national legislation and individual radiology departments and imaging facilities (involving the use of ionising radiation) must comply, providing evidence of this during any inspections by authorised external agencies. As mentioned earlier, undertaking clinical audit “in accordance with national procedures” is mandated within the Directive with clinical audit processes also subject to inspection as part of evaluation of BSSD compliance. Although not directly inspected per se, regulatory audit (and re-audit) to ensure compliance with specific BSSD requirements is clearly good practice for radiology departments and other departments with imaging facilities (involving the use of ionising radiation). Comprehensive explanation of clinical and regulatory audit processes, the differences between them and their role in radiation protection is provided in recent European Commission and Heads of the European Radiological Protection Competent Authorities (HERCA) publications [3, 6].

The 2021 BSSD uptake survey was, as in 2018, directed to the ESR EuroSafe Imaging Star Network. The survey response rate (61%, 78/128 departments) was good and comparable to the 2018 survey (64%, 66/103), making significant response bias unlikely. The co-operation of a large number of radiology departments in completing survey returns during difficult times is acknowledged and appreciated. These departments, by virtue of their involvement in the EuroSafe Imaging Star initiative and their wide geographic distribution, are likely to be representative of wider European radiological practice and these departments also have access to relevant ESR radiation protection and audit-related initiatives and publications.

The 2021 survey was distributed as a follow-up survey, following the 2018 pilot-formal standards/targets were again not included. As noted previously in 2018, all BSSD requirements covered in the survey are fixed and compulsory with a target of 100%. The need for regulatory audit and re-audit is not an BSSD requirement, but embedding effective processes of regulatory audit has clear benefits for radiology departments as documented earlier and can be considered good-practice.

Some general observations can be made on evaluation of the survey results: -

- Supporting processes of clinical audit are required by the BSSD and would be subject to formal inspection. The survey only briefly touched on clinical audit, with the opening question relating to departmental presence of a clinical audit infrastructure to support BSSD implementation. There was a reduction in positive responses to this question when compared to 2018 (47/70.15% in 2021, 54/81.82% in 2018), noting a higher % of departments answering “in development” in 2021 (10.61% vs 19.40%).
- In terms of implementation of specific BSSD parameters a mixed picture of both improvement and deterioration in compliance emerges. A significant improvement in compliance is evident between the two surveys in relation to the survey question relating to the BSSD dose limits and occupational exposure to the eye (2018 57.63%, 2021 80%).
- A similar mixed pattern of responses is present in relation to those questions referring to having a process in place to audit regulatory requirements. Quite marked improvement is seen since 2018 in those questions relating to dose limits to eye/skin for apprentices/students, with significant worsening in compliance identified for having a written protocol in place and training documented where justification is delegated to non-radiologists.
- In most cases an improvement since 2018 is present for questions covering regular re-audit of compliance with BSSD requirements (noting a relatively low yes/no response rate for this section in 2021).
- An additional important finding is the higher proportion of “skipped” responses provided in the 2021 survey compared to 2018. The increased tendency for “skipping” is a constant finding throughout the 2021 results and is particularly in evidence in relation to the audit/re-audit questions. The reasons for this are unclear – however the BSSD requirements covered in this survey are of relevance to most radiology departments and it is likely that in a proportion of “skipped” responses this actually translates into a failure of implementation. If this were to be the case then clearly this would have an adverse effect on reported departmental compliance in 2021 and comparisons with 2018 would be affected.

As was mentioned in the 2019 paper reporting the results of the 2018 survey [4] caution should be applied when drawing conclusions from survey data in terms of accuracy and generalisability. The work in progress observed in the departmental responses from 2018 has not materialised uniformly and the overall impression from the 2021 survey is that of persistently variable compliance amongst European radiology departments in terms of BSSD implementation, despite a number of ESR (and other organisation) radiation protection and audit related initiatives.

The most obvious explanation for the 2021 survey findings is the COVID-19 pandemic. Since early 2020 COVID-19 has caused significant problems for European (and worldwide) healthcare systems. Radiology departments and their staff have been at the forefront of dealing with these patients, radiology staff often becoming patients themselves. Radiology staff may have been redeployed to other areas, staff working offsite will have reduced availability and face-to-face meetings, audit sessions etc. will have been cancelled. It is easy to see how this crisis will have diverted attention and resources away from the more mundane and everyday aspects of radiology departmental practice and procedure. A proportion of departments may have overestimated their ability to implement necessary infrastructure and process changes when questioned in 2018 and COVID subsequently required re-allocation of available resources.

Embedding effective processes of clinical audit, radiation protection and clinical audit in medical radiological practices (those involving exposure to ionising radiation) are key priorities for the European Commission, other European and international agencies and also the professional societies. A number of key ESR initiatives were either in place prior to the 2018 survey or introduced subsequently. However, the potential benefits of these initiatives are likely to have been disrupted by the COVID-19 pandemic before they had time to influence radiology practice at departmental level. The ESR published Esperanto – A Guide to Clinical Audit and Clinical Audit Tool, in 2017 [7, 8]. An enhanced version 2 of Esperanto was launched at the European Congress of Radiology (ECR) in 2019. Esperanto is designed to support radiology departments in developing an effective programme of clinical audit, one that also supports BSSD compliance. Esperanto is in 2 sections, an initial guide explaining the principles and definitions of clinical audit and its relationship to radiation protection and the BSSD, the second section containing a series of audit templates for departmental use and adaptation as appropriate. The templates have a focus on regulatory audit but there is a clinical audit section in addition. Esperanto is currently under further development, with the launch of version 3 planned for ECR in 2022. The new version will have expanded sections covering clinical audit and also current best practices and guidances. Overall its emphasis will shift towards clinical audit in the template section, although regulatory audit templates will be maintained. New templates covering non-radiation protection related clinical audit and also clinical audits which specifically support BSSD compliance will be introduced. An updated ESR application process for membership of the EuroSafe Imaging Star Network is currently in preparation, this will include an increased emphasis on demonstration of compliance with BSSD requirements. Alongside Esperanto, ECR features bespoke sessions covering and promoting clinical audit with guidance on best practice and explanation of core principles. The EuroSafe Imaging Call for Action, 2018 [5] is a keynote ESR flagship campaign promoting and strengthening quality and safety in medical imaging and clinical audit is an important component.

It is also appropriate to mention the QuADRANT initiative at this juncture [9]. In 2019 the European Commission (EC) put out at tender N^o^ ENER/D3/2019–231-2 “Constant improvement in quality and safety of radiology, radiotherapy and nuclear medicine through clinical audit”. The ESR, as lead of a consortium also involving the European Association of Nuclear Medicine (EANM) and European Society of Radiotherapy and Oncology (ESTRO), was awarded the tender. The acronym QuADRANT was adopted (Quality Improvement Through Clinical Audit in Diagnostic (including Interventional) Radiology, Radiotherapy and Nuclear Medicine including Therapies). QuADRANT has core aims: -

- To review the status of implementation of clinical audits in the Member States.
- To identify good practices in Member States and available guidance and resources for clinical audits at national, European and international level.
- To provide further guidance and recommendations on improving the implementation and integration of clinical audits into national healthcare systems.
- To identify the potential for further co-ordinated EU action on quality and safety of radiology, radiotherapy and nuclear medicine.

QuADRANT commenced in January 2020 and will run over 30 months, the project includes two conferences and a main survey on European clinical audit practice, process and infrastructure and involving key players in European healthcare and clinical audit. This is a very important piece of work, looking to create a roadmap of clinical audit best practice with an emphasis on clinical audit in support of radiation protection.

Importantly, also in 2021, the European Commission announced the action plan for the SAMIRA (Strategic Agenda for Medical Ionising Radiation Applications) initiative [10]. One of the three major elements of SAMIRA is the European Initiative on Quality and Safety of medical applications of ionising radiation, designed to ensure that diagnostic and therapeutic uses of ionising radiation in European Member States conform to the highest standards. SAMIRA will support the incorporation of clinical audit practices into Member States’ healthcare systems. One action planned under SAMIRA involves seeking to improve justification of imaging use of ionising radiation, in line with European Council conclusions on this topic published in 2015 [11] Clinical audit will play a significant part in implementing this improvement. The ESR welcomes the SAMIRA Action Plan, and looks forward to collaborating with the European Commission and other stakeholders in bringing it to fruition.

The previous publication covering the 2018 BSSD uptake survey [4] did highlight the need for collaboration between relevant European agencies, national governmental bodies and national professional societies to facilitate improved BSSD uptake across Europe amongst European radiology departments. The ESR has undertaken additional surveys (pre-COVID-19) evaluating both the current state of clinical audit practice amongst European National Radiological Societies and also obtaining feedback on Esperanto from European radiology departments [12, 13]. These surveys demonstrated marked variability in clinical audit infrastructures, BSSD compliance and awareness of ESR-related audit initiatives. A core requirement for improving clinical audit uptake and BSSD compliance in European radiology departments will be establishing functional national infrastructures across all Member States, with the National Radiological Society networks well placed alongside the ESR and other specialist and healthcare bodies to facilitate necessary change.

## Conclusion

This re-survey of EuroSafe Imaging Star Departments evaluating BSSD uptake and supporting audit processes has demonstrated a mixed pattern of improvement and deterioration, with reduced compliance in many areas, since the previous 2018 survey. COVID-19 has caused significant service disruption between the two surveys and may explain, at least in part, the lack of consistent improvement that might have been anticipated in light of subsequent clinical audit/radiation protection initiatives by the ESR and other bodies. The ESR, working with partner organisations including the European Commission, will continue to promote clinical audit uptake and implementation. QuADRANT, led by the ESR, and SAMIRA are key European Commission initiatives and will help drive improvements in patient safety, experiences and outcomes, both in radiology but also in other medical specialties involved in the utilisation of ionising radiation.

## Supplementary Information

**Additional file 1: Table.** Pilot Survey on uptake of European BSS Directive (2013/59 Euratom) requirements with particular focus on clinical audit in European radiology departments.

## Data Availability

Data reporting the results reported in the article can be found in the form of tables which are accessed within the article.

## Abbreviations

BSSD: Basic Safety Standards Directive
EANM: European Association of Nuclear Medicine
ECR: European Congress of Radiology
ESR: European Society of Radiology
ESTRO: European Society of Radiotherapy and Oncology
EU: European Union
HERCA: Heads of the European Radiological Protection Competent Authorities
SAMIRA: Strategic Agenda for Medical Ionising Radiation Applications
